# Long-term High Resolution Image Dataset of Antarctic Coastal Benthic Fauna

**DOI:** 10.1038/s41597-022-01865-7

**Published:** 2022-12-03

**Authors:** Simone Marini, Federico Bonofiglio, Lorenzo Paolo Corgnati, Andrea Bordone, Stefano Schiaparelli, Andrea Peirano

**Affiliations:** 1 National Research Council of Italy (CNR), Institute of Marine Sciences, La Spezia, 19132 Italy; 2 Stazione Zoologica Anton Dohrn, Naples, 80121 Italy; 3 ENEA-Marine Environment Research Centre, La Spezia, 19132 Italy; 4 DISTAV, Università di Genova, Genova, 16132 Italy

**Keywords:** Biodiversity, Biodiversity

## Abstract

Antarctica is a remote place, the continent is covered by ice and its surrounding coastal areas are frozen for the majority of the year. Due to its peculiarity the observation of the underwater organisms is particularly difficult, complicated by logistic factors. We present a long-term dataset consisting of 755 images acquired by using a non-invasive, autonomous imaging device and encompassing both the Antarctic daylight and dark periods, including the corresponding transition phases. All images have the same field of view showing the benthic fauna and part of the water column above, including fishes present in the monitored period. All the images are manually annotated after a visual inspection performed by expert biologists. The extended monitoring period and the annotated images make the dataset a valuable benchmark suitable for studying the dynamics of the long-term Antarctic underwater fauna as well as for developing and testing algorithms for automated image analysis focused on the recognition and classification of the Antarctic organisms and the automated analysis of their long-term dynamics.

## Abstract (summary)

**Table:** 

Measurement(s)	Coastal Benthic Megafauna
Technology Type(s)	Autonomous Imaging Device
Factor Type(s)	None
Sample Characteristic - Organism	Sterechinus neumayeri • Odontaster • Trematomus • Polynoidae • Diplasterias • Ophiuridae • Ammothea • Neobuccinum eatoni • Staurocucumis • Flabegraviera mundata
Sample Characteristic - Environment	coastal sea water
Sample Characteristic - Location	East Antarctica

## Background & Summary

Antarctica is one of the most remote and inaccessible places on the Earth. The continent is constantly covered by ice and its surrounding coastal areas are frozen for the vast majority of the year^1^. Due to its peculiarity, the Antarctic continent and the corresponding marine environment are extremely sensible to both climate changes and anthropic impacts^2,3^. For this reason the need of long-term monitoring actions is strongly supported by the scientific community^2–6^ and by the international committees steering the Antarctic research^7–9^.

Many studies collect and analyze Antarctic long-term datasets dealing with benthic fauna, fishery and plankton communities^3,10–12^, while other studies investigates ice-sheet dynamics, iceberg tracking and meteorological data^13–18^.

Long-term time series are particularly important as they disclose relevant information on the temporal variation of the environment and of the living communities. This is true especially for the benthic fauna, where systematic observations repeated in time provide information useful for a detailed understanding and predictions of their dynamics. Furthermore, structure and composition of the benthic fauna are used as an effective tool to identify the impacts of environmental factors as well as the impact of human activities^5,6,19–21^.

Underwater video-monitoring methodologies are a consolidated approach for the ecosystem investigation^22–24^. Their action is non-destructive and the acquired data provide a highly informative content about the observed organisms^19,25,26^. Despite its effectiveness underwater video monitoring in the Antarctica regions is rarely practiced. Examples of short-term video monitoring activities are the photogrammetry approach proposed in^6,20^, where seabed footage was used for producing 3D models of the sea floor for identifying and counting the lying organisms. Other examples rely on time-lapse cameras deployed for few days in coastal areas, for observing benthic organisms^27,28^.

Within this context, this article describes the image dataset acquired within the ICE-LAPSE project^29^ through a long-term autonomous video monitoring activity^30^ continuously performed from January to November 2017 near the Italian Antarctic station Mario Zucchelli (Terra Nova Bay, Ross Sea).

The image dataset was acquired by using a stand-alone imaging device, specifically conceived for autonomous deployment extended in time^30–33^, positioned on the seabed of a coastal area at 20 m depth. The dataset consists of 755 images acquired every nine hours, encompassing both the Antarctic daylight and dark periods, including the corresponding transition phases. The imaging device was deployed onboard a fixed lander, all the images have the same field of view and show a sponge *Mycale (Oxymycale) acerata* Kirkpatrick, 1907 and its surrounding area including the corresponding fauna. The acquired dataset was visually inspected by expert biologists and the organisms present in each image were manually tagged for a total of 23881 individuals belonging to 12 different taxa.

The monitoring period extended in time and the tagged images make the dataset a valuable benchmark suitable for studying the dynamics of the Antarctic benthic fauna as well as for developing and testing algorithms for automated image analysis focused on the recognition and the classification of the Antarctic organisms and the automated analysis of their long-term dynamics.

## Methods

### Study area

**Fig. 1:**
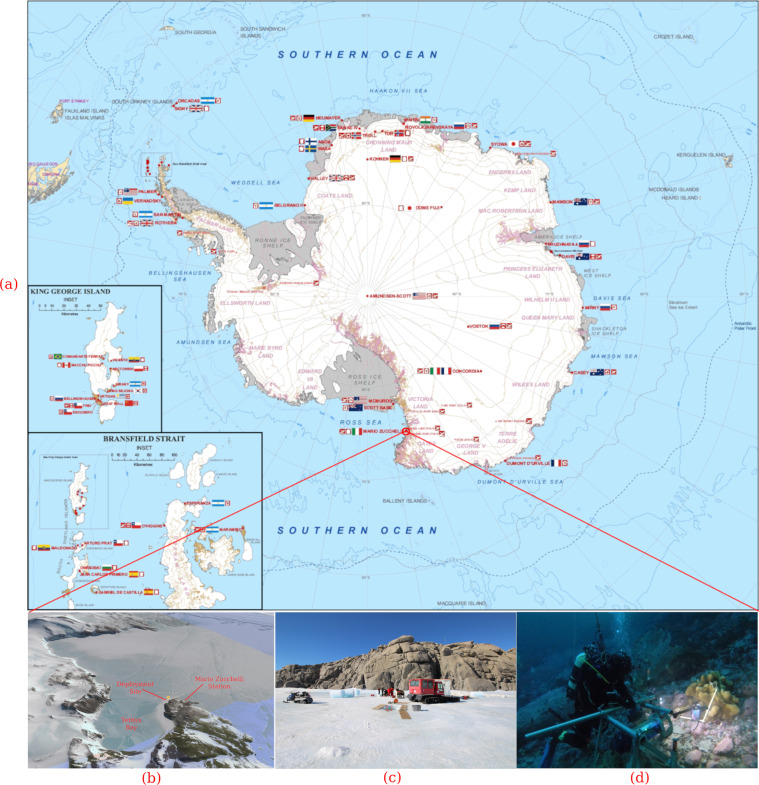
Overview of the monitoring activity near Mario Zucchelli Station, Terranova Bay (Ross Sea) - 74° 41.410′ *S*, 164° 06.233′ *E* (**a**) and (**b**); the deployment site on the frozen sea surface (**c**) and at the seabed (**d**).

The underwater images were acquired in Tethys Bay, a coastal area near the Italian Antarctic station Mario Zucchelli (74° 41.410′ *S*, 164° 06.233′ *E*), as shown in Fig. 1a,b.

Tethys Bay is an inlet 1600m wide and 3000 m long, oriented to Northwest. It is surrounded by glaciers and granite cliffs and its maximum depth is about 280 m in the central part. The sea pack in the bay persists on average from the end of February to the beginning of January^6,34,35^. The monitored site, known as “Zecca”, is located in front of a granite cliff as shown in Fig. 1c. The seabed at the “Zecca” site is characterized by a moderate slope composed of pebbles and scattered with large stone blocks as shown in Fig. 1d. The site, was selected because it is suitable for long term experiments as the seabed is protected from icebergs and wave storms.

The monitored area is rich of sponges *Mycale (Oxymycale) acerata* Kirkpatrick, 1907, sea urchins *Sterechinus neumayeri* (Meissner, 1900) and sea stars, mainly *Odontaster validus* Koehler, 1906 and *Diplasterias brucei* (Koehler, 1907) present all over the year, while fishes and several benthic organisms show different behaviors depending on the Antarctic season^30^.

The average duration of daytime at the Mario Zucchelli Station was estimated and discussed in^36^, resulting that from 3rd November to 7th February the sun is above the horizon for 24 hours per day continuously, while from 3rd May to 10th August natural light is completely absent. The natural light dynamics in Antarctica, are one of the parameter driving the faunal dynamics^37,38^ and the diffused light reaching the seabed through the icepack is an important factor conditioning the benthic fauna. The accumulation of snow on the sea pack or the shadow projected by cliffs surrounding the studied area can locally change the underwater diffused light dynamics and in the period in which the sun is constantly present above the horizon, the monitored site shows a daily light/shadow cycle. As discussed in^28,30^, the shadow effect is caused by the position of the cliff with respect to the monitoring site and such a light variation has been captured by the acquired images.

### Image acquisition device

The image dataset was acquired by an imaging device based on the European patent EP 2863257 B1^31^. The imaging device was installed on a metal frame deployed through a whole practiced in the sea pack (Fig. 1b) and secured by divers with metal pins and ballasts on the seabed at 20 m depth, distant about one meter from a sponge *M. acerata* (Fig. 1d). Though the technology described in the patent can execute on board image processing algorithms and perform data communication^39^, the device deployed for acquiring the presented dataset was designed only for acquiring and storing underwater images, without any communication and image processing features.

**Fig. 2:**
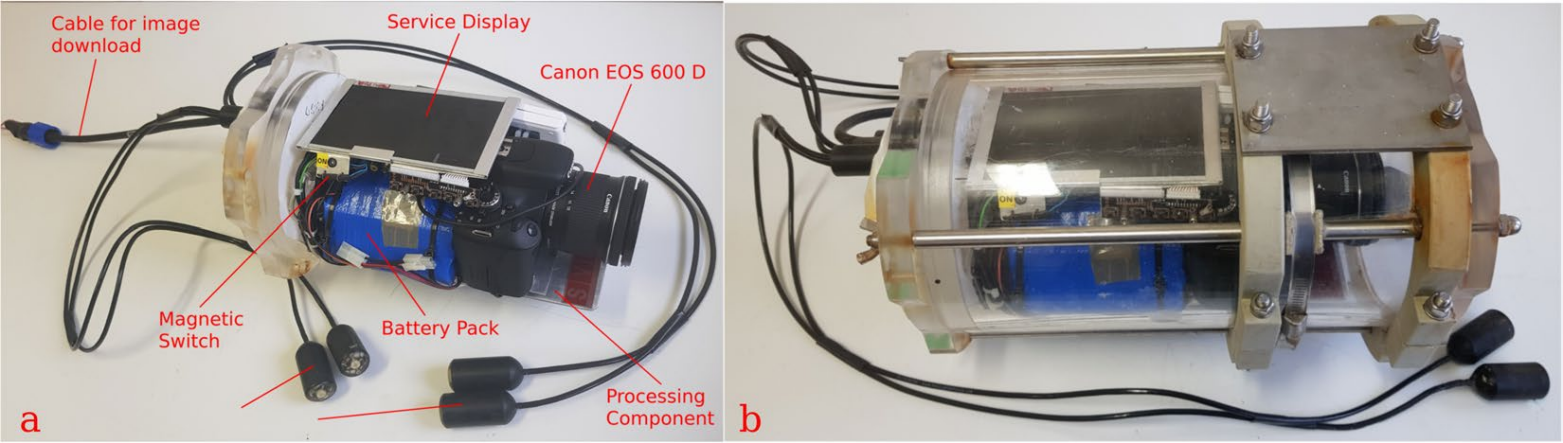
The autonomous and stand-alone imaging device used for the image acquisition and its main hardware components (**a**); The imaging device inside its transparent underwater case (**b**).

The imaging device was based on a commercial Canon EOS 600D reflex camera whose behavior was controlled by a firmware based on the Magic Lantern toolkit^40^ and installed on the camera for the automated acquisition of the image dataset. The customized firmware was aimed at the automatic shooting of the images and, during the deployment activities performed by divers, at redirecting a video stream to a service display used for the correct positioning of the camera (see Fig. 2).

The optical group used for acquiring the images was a CANON Zoom Lens EF-S 10–18 *mm* (1:4.5–5.6 IS STM ϕ67 *mm*), set with focal length equal to 12 *mm*. The lighting system consist of four high performance LEDs, (3.4 *V*,1 *W*,350 *mA*,75 *lm*, cold white color) embedded in epoxy resin and wired to the device underwater case, as shown in Fig. 2a. The four LEDs were split into two groups (two LEDs each group) each one displaced about 40 *cm* from the device and oriented toward the sponge in front of the lander as shown in Fig. 1d. In order to position the imaging device correctly in front of the sponge, a service display was installed inside the transparent underwater case (Fig. 2) aimed at providing a live stream of the subject framed by the camera. The overall behavior of the imaging device was defined by a control hardware component managed by a diver operator through a magnetic-switch that allowed to switch between the live view stream and two operative modes for the image acquisition^30^.

The imaging device was powered by a main battery pack consisting of nine primary batteries Tadiran *SL*-2700 Series (3.6 *V*, 35.0 *Ah*, size DD) coupled with a secondary rechargeable battery pack consisting of four Samsung 18650 batteries (3.6 V, 3450mAh, max discharge current 8 A). The main battery pack was used for providing the energy needed for the long term monitoring action, while the secondary battery pack was used for supplying the impulse of power needed for switching on the camera and the lighting system.

More details on the imaging device used for acquiring the Antarctic image dataset are provided in^30^, while other monitoring activities using a similar technology can be found in^32,33,41^.

## Data Records

**Fig. 3:**
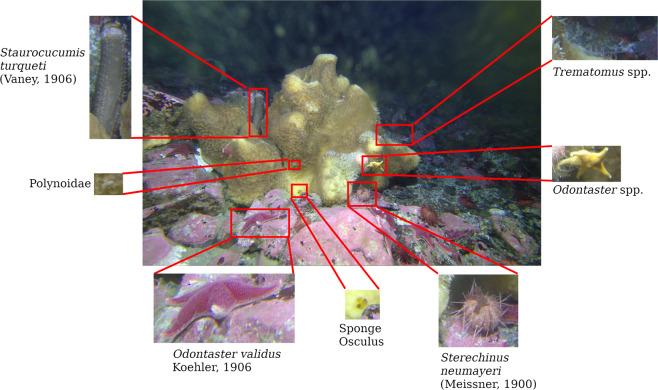
Example of underwater image acquired by the imaging device. Detailed examples of the present faunal organisms are highlighted.

The dataset consists of 755 high resolution color images acquired every nine hours from 24 January 2017 to 15 November 2017. Each image has a resolution of 5207 × 3469 pixels and was saved in the Canon proprietary raw format CR2^42^. The collected images were visually inspected and the framed organisms were counted, tagged and labeled. The Fig. 3 shows an example of acquired image highlighting some organisms captured by the imaging device.

**Fig. 4:**
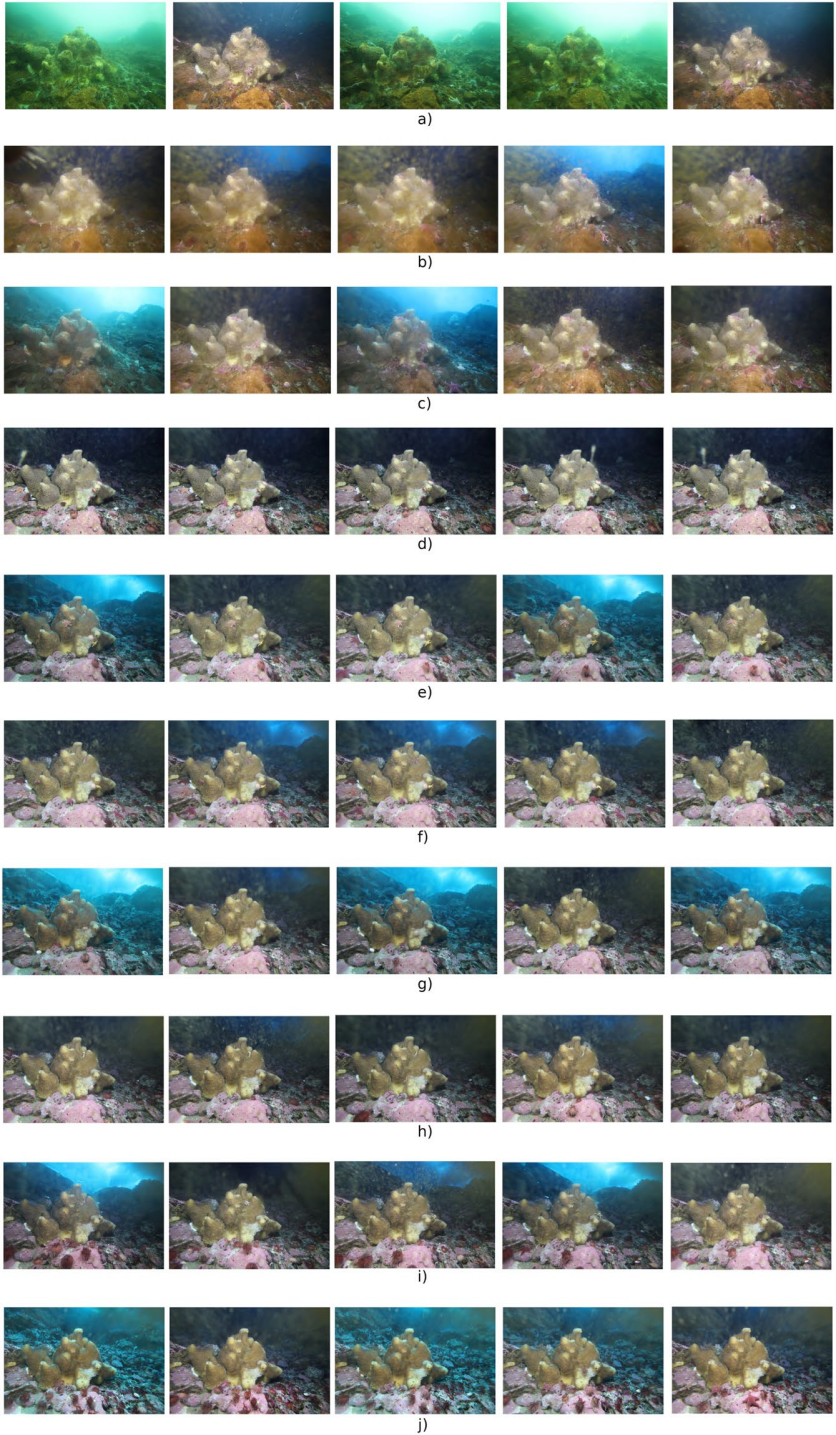
A set of examples sampled form the image dataset. Each row consists of images acquired consecutively, while different rows correspond to different periods within the monitoring activity.

The image dataset encompasses different environmental conditions including short-term and seasonal light dynamics, icepack coverage and a short period with occurrence of bio-fouling on the camera porthole. The images shown in Fig. 4 provide several examples of the environmental conditions occurring along the acquisition period of the image dataset. Each row contains five images acquired consecutively, while different rows correspond to images sequences acquired in different periods and spanning the whole image dataset.

The images in Fig. 4a–c exemplify the acquisitions between the end of February and the beginning of May. Each of the three sequences alternate bright images, where the natural ambient light is diffused through the water column (and the icepack when present), and darker images corresponding to the dark short-term periods produced by the light/shadow cycle caused by the cliff in proximity of the monitored site^28,30^. During the shadow periods the natural diffused light is attenuated and the foreground objects (e.g. the sponge) are illuminated by the lighting system of the imaging device. The water’s greenish hue in Fig. 4a, acquired in February, is caused by a huge phytoplankton bloom occurring after the icepack melting. In the sequence shown in Fig. 4b, corresponding to march, the icepack is present again and the strong reduction of diffused light might be probably caused by a temporary cloudy sky or by the accumulation of snow on the icepack surface. The images in Fig. 4b also show the presence of bio-fouling on the porthole of the camera, following the phytoplankton bloom. Such bio-fouling is suddenly reduced from the sequence in Fig. 4c, corresponding to April, until the end of the acquisition period (Fig. 4j), corresponding to November.

Examples of images acquired during the Antarctic night are shown in Fig. 4d, corresponfing to June. In this case the bio-fouling is not present anymore, the diffused natural light is completely absent and all the images in the sequence are illuminated by the lighting system of the imaging device. After the end of the Antarctic night (end of August) the natural light increases with some discontinuities, Fig. 4e–g, until the end of September where a significant decrease happens as shown in Fig. 4h–j, yet probably caused by a cloudy sky or by the accumulation of snow on the sea pack. In all the images from Fig. 4b to Fig. 4j it is possible to note the presence of the frozen sea surface, during the daylight periods.

After the acquisition of the first 109 images (out of 755 images), the imaging device moved slightly downward and rightward, producing an offset of a few pixels (30 × 108 pixels) in the position of the sponge with respect to the borders of the image.

The image dataset is accessible through the Zenodo repository^43^ and consists of the records presented in Table 1.

**Table 1:** The image datset archives.

Filename	Content	Size
Raw_Images.tgz	Raw images with 5207 × 3469 pixels resolution in CR2 format	18.6 *GB*
JPG_Images.tgz	Images with 5207 × 3469 pixels resolution in JPEG format	2.0 *GB*
640 × 427.tgz	Images with 640 × 427 pixels resolution in JPEG format	72.3 *MB*
ICE-LAPSE_tags.tgz	Information on the organisms’ tags and species	2.7 *GB*
iFDO_Dataset.tgz	Images to be used within the iFDO standard^45^	2.0 *GB*
Deployment in Tethys bay - Mario Zucchelli Station - 2015_GUARD1-PNRA-2015_iFDO.yaml	iFDO metadata^45^	359 *KB*

All the images in the archives Raw_Images, JPG_Images, 640 × 427 and ICE-LAPSE_tags have a filename starting with the string IMG _<*id*>, where <*id*> is a number varying between 609 and 1367 that unambiguously identifies each image file. The two archives Raw_Images and JPG_Images contain the full resolution images in raw and JPEG formats, respectively. The ICE-LAPSE_tags archive provides information on the organisms classification performed by expert biologists. It contains two folders and three files:

- TaggedImages
- TextualTags
- Classes.txt
- SpeciesColorLabel.jpg
- SpeciesDistribution.csv

**Fig. 5:**
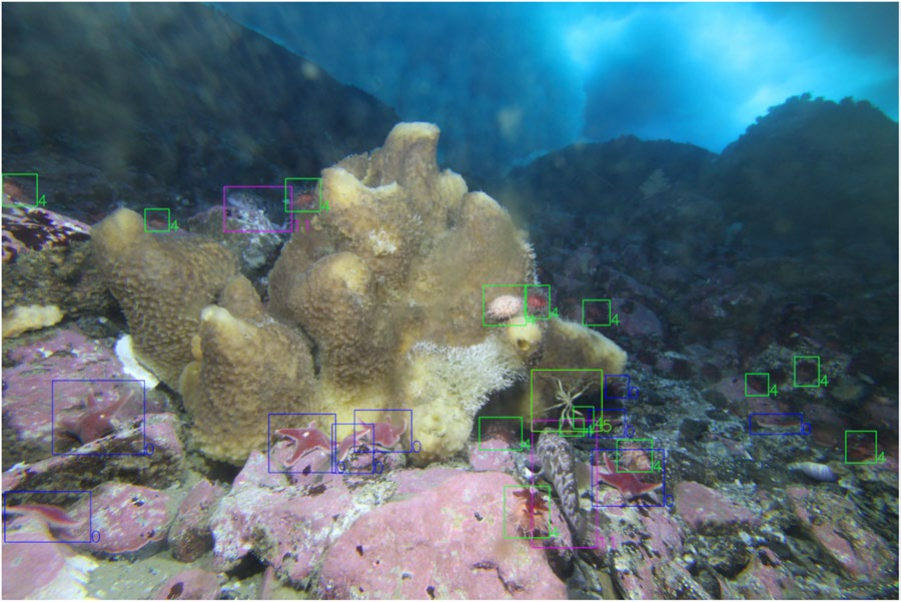
An example of tagged image, where the labelled (colored) boxes represent the following taxa: 0 *Odontaster validus* Koehler, 1906; 4 *Sterechinus neumayeri* (Meissner, 1900); 5 *Ammothea* sp.; 11 *Trematomus* spp.

The TaggedImages folder contains the dataset images where each organism identified in the visual inspection is highlighted by a colored bounding-box (tag) accompanied by a number. The color and the number associated to each tag represent the organism’s species as listed in the files Classes.txt or SpeciesColorLabel.jpg. An example of a tagged image is shown in Fig. 5.

The TextualTags folder contains one text file for each image of the dataset. Each row in the file represents a tag in the corresponding image. The image tagging has been performed with the labelImage^44^ graphical tool and each row in the file has the following format:

$$ < label\_id >  < x >  < y >  < w >  < h > ,$$

where <*label_id*> is a number identifying the species of the tagged organisms, as listed in the file Classes.txt, <*x*>, <*y*>, <*w*> and <*h*> are the coordinates of the centre of the tagging box and the corresponding width and hight, respectively. The file SpeciesDistribution.csv is an abundance matrix per species where the first column contains the time-stamp of each image, the second column contains the image indices and the remaining thirteen columns contain the number of individuals occurring in the images for each species.

The archive iFDO_Dataset.tgz contains the image dataset formatted according to the image FAIR Digital Object (iFDO) standard^45^, whose metadata are encoded in the file “Deployment in Tethys bay - Mario Zucchelli Station- 2015_GUARD1-PNRA-2015_iFDO.yaml”. Each image of the iFDO dataset is characterized by a universally unique identifier (uuid) and the corresponding SHA-256 image encoding. The metadata encoded in the yaml file contain general information about the whole dataset (e.g. the dataset uuid, dataset abstract, dataset authors, type of illumination, type of instrument used) and specific metadata for each image (e.g. depth, latitude, longitude, altitude, uuid, the SHA-256 hasing code).

Finally, each dataset image, either in raw or JPEG format, contains the corresponding EXIF data, encoding several image acquisition information like for example time exposure, iris aperture, focal length and ISO, together with other low level metadata.

## Technical Validation

All the images in the dataset were visually inspected and all the animals in the images were annotated using the labelImg software tool^44^.

**Fig. 6:**
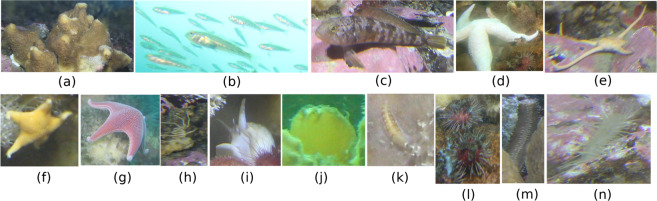
Examples of species present in the image datset as reported in^30^. (**a**) *Mycale (Oxymycale) acerata* Kirkpatrick, 1907; (**b**) *Trematomus newnesi* Boulenger, 1902; (**c**) *Trematomus* sp.; (**d**) *Diplasterias brucei* (Koehler, 1907); (**e**) Ophiuridae; (**f**) *Odontaster* sp.; (**g**) *Odontaster validus* Koehler, 1906; (**h**) *Ammothea* sp.; (**i)** *Neobuccinum eatoni* (E. A. Smith, 1875); (**j**) Unclassified; (**k**) Polynoidae; (**l**) *Sterechinus neumayeri* (Meissner, 1900); (**m**) *Staurocucumis turqueti* (Vaney, 1906); (**n**) *Flabegraviera mundata* (Gravier, 1906).

In order to avoid any ambiguity in the classification of organisms, the dataset was independently inspected by two expert biologists. The inspection activity produced the list of taxa shown in Fig. 6, that was used by a third operator to annotate the images. In the event of lack of agreement between the two experts, the genus or the family was used to classify an individual.

The visual inspection resulted in to a total of 23881 organisms visually classified and tagged, as summarized in Table 2. The fauna classification shown in the table is based on the scientific literature describing the fauna present in the area of Mario Zucchelli Station. This classification is supported by the continuous samplings and taxonomic analysis performed in the last twenty years^5,46–48^.

**Table 2:** Distribution of image tags per species, as reported in^30^.

Species	Number of Tags
*Sterechinus neumayeri* (Meissner, 1900)	16605
*Odontaster validus* Koehler, 1906	5965
*Trematomus newnesi* Boulenger, 1902	401
*Trematomus* spp.	267
Polynoidae	143
*Odontaster* spp.	139
*Diplasterias brucei* (Koehler, 1907)	137
Ophiuridae	79
*Ammothea* sp.	70
*Neobuccinum eatoni* (E. A. Smith, 1875)	23
Unclassified	21
*Staurocucumis turqueti* (Vaney, 1906)	17
*Flabegraviera mundata* (Gravier, 1906)	14
**Total tags**	**23881**

Figure 6 shows some examples of the 14 taxa present in the acquired images.

Several analysis on the image dataset were presented and discussed in^30^. The manual tags were used to investigate the long- and mid- term trends of the organism abundance, as well as the single species distribution along the monitored period. The abundance trends capture the overall correlation between the seasonal trend of the natural light and the behavior of the benthic fauna, together with short-term fluctuation of the individual counts suggesting more complex faunal behavior driven by environmental parameters independent by the underwater diffused light. The single species temporal distribution shows that different species have different behaviors, depending on the seasonal characteristics, as reported in^30^.

Also automated image analyses have been performed on the proposed image dataset. A computer vision based approach was used for estimating the underwater diffused light from the acquired images. The obtained results are coherent with the average daylight duration at Mario Zucchelli Station discussed in^36^ and highlight the light/shadow cycle reported in^28^. Other automated analyses reported in^30^ deal with the movement estimation of the organisms in proximity of the framed sponge, the analysis of the temporal dynamics of the sponge’s oscula and the use of the YOLO Convolutional Neural Network^49,50^ for automatically detecting sea stars and sea urchins.

## Data Availability

The image dataset was tagged by using the labelImage^44^ graphical tool.
